# *Lactobacillus fermentum* 3872 as a potential tool for combatting *Campylobacter jejuni* infections

**DOI:** 10.1080/21505594.2017.1362533

**Published:** 2017-08-25

**Authors:** B. Lehri, A. M. Seddon, A. V. Karlyshev

**Affiliations:** The School of Life Sciences, Pharmacy and Chemistry, SEC Faculty, Kingston University, Kingston Upon Thames, UK

**Keywords:** adhesion, antimicrobials, *Campylobacter jejuni*, collagen I, collagen binding protein, *Lactobacillus*, multi-drug resistance, probiotics

## Abstract

Due to the global spread of multidrug resistant pathogenic bacteria, alternative approaches in combating infectious diseases are required. One such approach is the use of probiotics. *Lactobacillus fermentum* 3872 is a promising probiotic bacterium producing a range of antimicrobial compounds, such as hydrogen peroxide and lactic acid. In addition, previous studies involving genome sequencing and analysis of *L. fermentum* 3872 allowed the identification of a gene encoding a cell surface protein referred to as collagen binding protein (CBP) (not found in other strains of the species, according to the GenBank database), consisting of a C-terminal cell wall anchor domain (LPXT), multiple repeats of ‘B domains' that form stalks presenting an “A domain” required for adhesion. In this study, we found that the CBP of *L. fermentum* 3872 binds to collagen I present on the surface of the epithelial cells lining the gastrointestinal tract. Moreover, we found that this host receptor is also used for attachment by the major gastrointestinal pathogen, *Campylobacter jejuni*. Furthermore, we identified an adhesin involved in such interaction and demonstrated that both *L. fermentum* 3872 and its CBP can inhibit binding of this pathogen to collagen I. Combined with the observation that *C. jejuni* growth is affected in the acidic environment produced by *L. fermentum* 3872, the finding provides a good basis for further investigation of this strain as a potential tool for fighting Campylobacter infections.

## Introduction

*Campylobacter jejuni* is an enteric pathogen and one of the most common causes of gastroenteritis in humans with symptoms such as abdominal pains, watery or bloody diarrhea, and fever.^1^ In rare cases, *C. jejuni* infections can lead to a neurodegenerative disease such as Guillain-Barre syndrome.^2^ *C. jejuni* infections are often caused by poor hygiene standards, consumption of undercooked meat, contaminated water and/or milk.^3^ Fatalities associated with *C. jejuni* infections are uncommon, although can occur among immunologically naïve patients.^4^ *C. jejuni* infections are an economic burden leading to many hospitalisations/primary care visits.^5^

There has been a rise in antimicrobial resistant forms of *C. jejuni* caused by the misuse of antimicrobials.^6^ *C. jejuni* has also been placed on a list of antibiotic-resistant priority pathogens by the world health organization (WHO) to promote research and development in novel antimicrobials.^7^ Due to the appearance of multidrug resistant forms of these bacteria, there is growing interest in alternative approaches to combat *C. jejuni* infections, such as those using probiotics, bacteriocins and bacteriophages, with the most recent focus on probiotics.^1^ Effective usage of the latter requires a better understanding of the molecular mechanisms of their action. The antagonistic activity of probiotics is associated with the production of bacteriocins, lactic acid, hydrogen peroxide, competition for nutrients and colonisation niches, as well as modulation of host immune response.^1,8^

*Lactobacillus fermentum* 3872 is a Gram-positive, facultative anaerobe isolated from milk of a healthy woman.^9^ *L. fermentum* 3872 produces lactic acid and hydrogen peroxide and is capable of binding to human HeLa and buccal cells.^9^ Genome sequencing of *L. fermentum* 3872 revealed genes required for bacterial survival in the gastrointestinal tract, as well as those potentially involved in interaction with fibronectin, mucin and collagen, such as the genes encoding enolase and 2 collagen binding proteins.^10,11^ The full and partial copies of the collagen-binding protein (CBP) encoding genes were found to be located on a plasmid and chromosome respectively.^10,11^ Collagen I, which is one of several types of collagens ubiquitous in mammals, is commonly found on the surface of host cells present in the gastrointestinal tract.^12^ In this study, we confirmed the affinity of the putative CBP of *L. fermentum* 3872 to collagen I and found that both CBP and *L. fermentum* 3872 compete with *C. jejuni* for binding to this host cell receptor. In addition, a role of *C. jejuni* flagellum in binding to collagen I was established.

## Results

### The purified CBP of *L. fermentum* 3872 interferes with *Campylobacter* binding to collagen I

**Figure 1.**
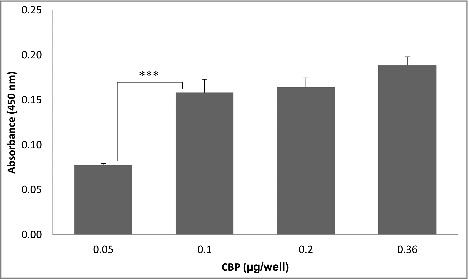
ELISA experiments showing CBP binding to collagen I; the data represent 2 biologic repeats each with 3 technical repeats (n = 6).

**Figure 2.**
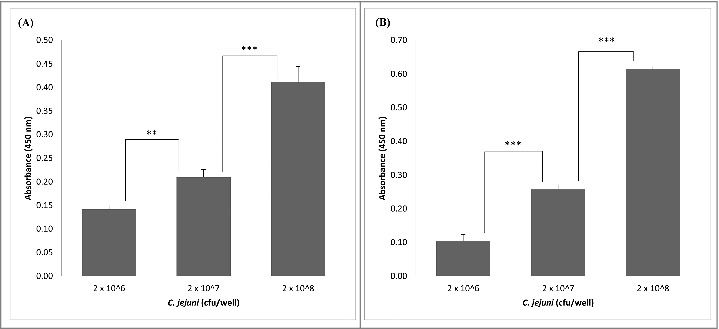
Detection of adhesion of *C. jejuni* strains 81–176 (A) and 11168H (B) to collagen I using ELISA; the data represent 2 biologic repeats each with 3 technical repeats (n = 6) apart from (A), 2 × 10^8^ cfu/well, where n = 5.

Expression of the *L. fermentum* 3872 CPB in *E. coli* as a His-tagged fusion protein allowed its purification as a stable product of an expected size (111 kDa predicted, 115 kDa estimated from a gel, Fig. S1). The slight (3.6%) difference in the sizes is likely to be due to conformational properties of the protein, which is typical for large outer membrane proteins of bacteria.^13^ It was found that the CBP binds to collagen I in a concentration-dependent manner with no increase in absorbance above 0.1 µg/well of CBP (Fig. 1). Since *C. jejuni* strains 11168H and 81–176 were also able to bind to collagen I in a concentration-dependent manner (Fig. 2), we aimed to establish if these bacteria compete with the purified CBP for the binding sites.

**Figure 3.**
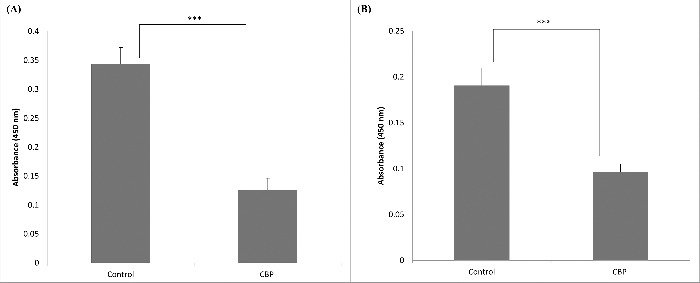
Effect of CBP (2 µg/well) on adhesion of *C. jejuni* strains 81–176 (A) and 11168H (B) in binding to collagen I, the bars labeled ‘control’ have respective *C. jejuni* strains (2 × 10^7^ cfu/well) only, while that labeled CBP has a mixture of 2 µg CBP and 2 × 10^7^ cfu/well *C. jejuni*; the data represent 3 biologic repeats each with 3 technical repeats (n = 9).

**Figure 4.**
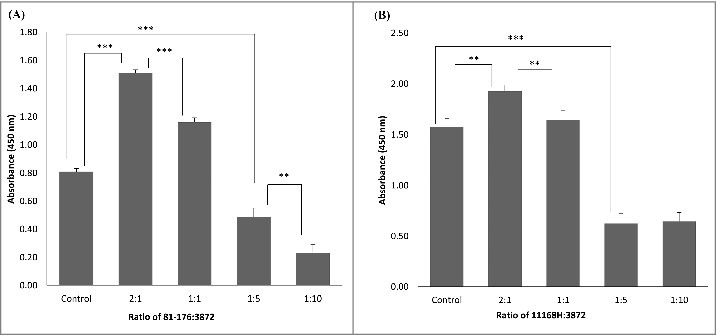
Competition between. *L. fermentum* 3872 and *C. jejuni* strains 81–176 (A) and 11168H (B) for binding to collagen I detected using ELISA, the bars labeled ‘control’ have respective *C. jejuni* strains of amount 2 × 10^7^ cfu/well added only, the ratios indicated on the graph are based on cell to cell amounts added to each well; the data represent 2 biologic repeats each with 3 technical repeats (n = 6). Control (B) and 1:10 (B) represent data with 3 biologic repeats each with 3 technical repeats each (n = 9).

Inhibition of *C. jejuni* attachment to collagen I was detected when using 2 µg of CBP per well (Fig. 3). We were then wondering if a similar inhibition of *Campylobacter* could be observed when using whole cells of *L. fermentum*. As expected, the inhibition was observed when using high *Lactobacillus*/*Campylobacter* cell ratios (Fig. 4). Surprisingly, some increase in *C. jejuni* binding was seen when using smaller amounts of *L. fermentum* 3872 (Fig. 4). As described in the discussion section, this could be a result of aggregation of *Campylobacter* bacteria.

### Identification of *C. jejuni* proteins involved in collagen I adhesion

**Figure 5.**
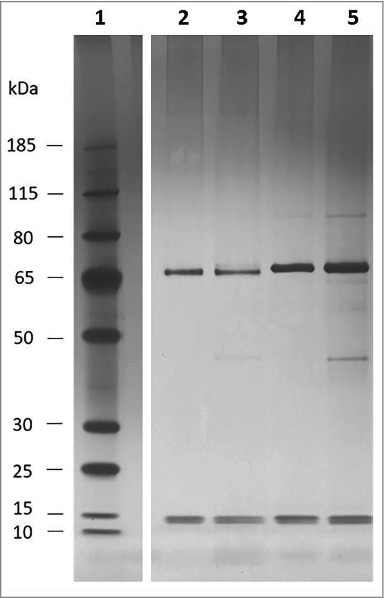
Silver staining of *C. jejuni* Co-IP eluate; 1, pre-stained ladder (Page ruler plus); 2, 11168H eluate after 1 hour incubation; 3, 11168H eluate after 3 hour incubation; 4, 81–176 eluate after 1 hour incubation; 5, 81–176 eluate after 3 hour incubation.

While genome sequencing reveals genes encoding potential collagen binding proteins of *L. fermentum* 3872, one of which was the subject of this study, no such proteins could be identified in the genomes of *C. jejuni* strains 11168H and 81–176. Therefore, attempts to identify such proteins were undertaken by using affinity binding followed by mass spectrometry (LC MS/MS). The analysis of the proteins bound to magnetic beads coated with collagen I revealed 2 major bands (65 kDa and 15 kDa, Fig. 5) in both strains tested

Analysis of these bands using mass spectrometry identified the top (65 kDa) bands in both strains as flagellin subunits (FlaA and FlaB). Larger observed sizes of these proteins, when compared with those predicted from their amino acid sequences (60 kDa), are likely to be a result of O-linked glycosylation.^14^ Indeed, it was reported that the molecular mass of flagellin of strain 81–176 as determined by gel electrophoresis was about 65 kDa, larger than that was predicted from its amino acid sequence.^15^ Slight difference in gel mobilities between flagellins from these 2 strains, as observed in Fig. 5 can also be explained by strain to strain variation in glycosylation pattern.

### *L. fermentum* 3872 inhibits *C. jejuni* growth by production of acidic environment

**Figure 6.**
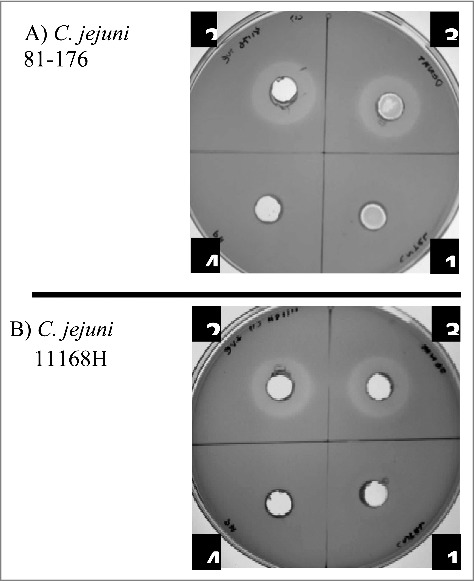
Inhibition of growth of *C. jejuni* strains 81–176 (A) and 11168H (B) in the presence of cell-free *L. fermentum* 3872 culture supernatant; 1, M.R.S broth (control); 2–4, *L. fermentum* 3872 cell-free supernatant; 2, untreated; 3, heat-treated; 4, pH adjusted. Three biologic repeats were performed.

Cell-free supernatants of *L. fermentum* inhibited the growth of *C. jejuni* (Fig. 6). Adjustment of the supernatant pH (normally about 4.2) to 6.3 abolished inhibition zone, suggesting that it was the acid environment that was causing the inhibitory effect. Acidification of the media is commonly attributed to the release of lactic acid by these bacteria. On the other hand, heat treatment had no effect on the inhibition zone (Fig. 6) indicating the absence of heat labile compounds involved in growth inhibition. The results suggest that the main anti-*Campylobacter* activity is associated with acidification of the environment.

## Discussion and conclusion

The results of this study suggest that *L. fermentum* and *Campylobacter jejuni* may exploit the same host cell receptor for attachment and colonisation. We demonstrated the molecular mechanism of such interaction and identified the adhesins required for binding of these bacteria to collagen receptor. As adhesion is important for *C. jejuni* host colonisation and infection,^16^ competition for adhesion to collagen I may be a viable means of reducing pathogen load in hosts and thus preventing *C. jejuni* infection. Interestingly, whole cell ELISA experiments indicated an increase in absorbance of *C. jejuni* being detected when using lower amounts of *L. fermentum* 3872. Potential reasoning for the latter observation could be explained by possible auto-aggregation of *C. jejuni* or co-aggregation between *C. jejuni* and *L. fermentum* 3872. Co-aggregation between *C. jejuni* and other species of *Lactobacilli* has previously been reported.^17^ *In vitro* experiments demonstrated that the presence of probiotics can lead to co-aggregation with *C. jejuni* and inhibition of adhesion of the latter to human epithelial cells.^18^

The ability of probiotic bacteria to cause aggregation of (or co-aggregation with) *C. jejuni* cells and inhibit their binding to host cells may work synergistically with other antibacterial factors. In particular, higher gastric acidity was found to reduce the likelihood of *C. jejuni* infection.^19^ This is supported by our study demonstrating that acidification of the environment caused by *Lactobacillus fermentum* represents a strong antibacterial factor. Co-aggregation may further assist in the antagonistic action of lactobacilli by reducing the distance between the probiotic cells and the pathogen.^17^ Utilization of multiple antagonisitic factors would elevate the effect of probiotics in inhibiting a pathogen, and reduce the risk of developing of antimicrobial resistance.

According to our data, *C. jejuni* flagellin binds to collagen I, supporting other data on the role of flagella in adhesion.^20,21^ To our knowledge, this is the first study on the identification of a host cell molecule specifically interacting with bacterial flagellum. The latter is known to be modified by O-linked glycosylation, which is variable in the same strain and is also strain-dependent.^22-24^

Due to the extreme variability of O-linked flagella modifications and the difference between the oligosaccharide structures decorating flagellins in the 2 strains tested, the involvement of sugar residues in binding to collagen seems unlikely.

It would be interesting to investigate other putative *L. fermentum* 3872 adhesins predicted from its genome sequence,^11^ such as enolase, mucus and fibronectin binding proteins, as well as aggregation substance precursor. The results of this study warrant further investigation of antagonistic activity of this strain in poultry. Due to its anti-campylobacter activity *L. fermentum* 3872 could potentially be used for prophylaxis of such *C. jejuni* induced diseases as traveler's diarrhea, inflammatory bowel disease and irritable bowel syndrome.^25,26^ Although it was isolated from human milk of a healthy person^9^ and is predicted to be generally safe, trial experiments are required to confirm its safety. Adhesion experiments using epithelial cell lines could be performed to determine competition between CBP or *L. fermentum* 3872 and *C. jejuni*. Furthermore, due to the presence of other genes encoding putative collagen-binding proteins such as enolase, *cbp* gene knockout experiments could be performed to determine the extent to which CBP plays a role in collagen adhesion for *L. fermentum* 3872. The experiments described in this study may also be conducted with other pathogenic bacteria, such as e.g. *Staphylococci* which utilize adhesion to collagen for host colonisation.^27^ With an increasing understanding of the mechanisms of interaction and competition between bacteria, a wide variety of tools may be developed for anti-microbial purposes, reducing our dependence on antibiotics and widening our means in combatting pathogenic bacteria such as *C. jejuni*.

## Materials and methods

### Bacterial strains and growth conditions

*L. fermentum* 3872 was grown overnight at 37°C under anaerobic conditions on de Man, Rogosa and Sharpe (M.R.S.) agar (Oxoid), and in M.R.S broth (Oxoid). *C. jejuni* 11168H is a hypermotile derivative of *C. jejuni* NCTC 11168 originally isolated from human faeces.^22^ *C. jejuni* 81–176 is a highly virulent strain isolated from raw milk.^28^ *C. jejuni* was grown for 24 hours at 37°C in a microaerobic incubator (Don Whitley Scientific) in an atmosphere of 10% CO_2_, 5% O_2_, N_2_ 85% on CBA (Columbia Blood Agar Base, Oxoid) supplemented with 5% defibrinated horse blood (Oxoid) and *Campylobacter* selective supplement Skirrow (Oxoid). *E. coli* was grown at 37°C overnight on LB (Luria Bertani) agar (Fisher Scientific) or in LB broth (Fisher Scientific) supplemented with chloramphenicol at 25 µg/ml where appropriate, e.g., for expression of CBP (see below).

### Cloning and purification of CBP

The *cbp* gene lacking the region corresponding to the leader peptide was PCR-amplified using the following primers: CBP_Forward, ATATGCTTCTAGAAGAAGGAGGCAACAGTATGCACCATCACCATCACCATGATAGCAAGACAAATATTACTCAGAACGGTACG and CBP_Reverse, ATGAGCATGCTCAAATAGTAAATCTACTTATAACTACTAAACC. The CBP_Forward primer contained a Shine-Dalgarno (SD) sequence, as well as a region encoding a hexa histidine tag. Polymerase chain reaction (PCR) was conducted by using a Q5 High-Fidelity DNA Polymerase kit (NEB) with the following conditions: 98°C for 30 seconds for initial denaturation, 25 cycles of denaturation for 10 seconds at 98°C, annealing for 30 seconds at 55°C and extension for 4 minutes at 55°C, and a final extension at 72°C for 2 minutes.

The PCR product was purified using the QIAquick PCR purification kit (Qiagen), digested with enzymes *Xba*I and *Sph*I (NEB) and cloned into expression vector pBAD33^29^ using Quick Ligation kit (NEB) and *E. coli* Express competent cells (NEB).

Sanger sequencing, conducted by GENEWIZ revealed no errors in the cloned fragment.

For protein expression, 10 ml of the overnight culture of bacteria containing the recombinant plasmid were inoculated into 250 ml of media, incubated at 37°C on a shaker at 120 rpm to OD_600_ of 0.6 and induced with L-arabinose (ACROS organics) at a final concentration of 0.1% for 3 hours. The protein was purified using a Clonetech His60 protein purification column. The concentration of protein was determined using a Pierce BCA protein assay kit.

Samples were analyzed on NuPAGE Novex 4–12% Bis-tris gel (ThermoFisher Scientific) after mixing with1X NuPAGE LDS sample buffer (ThermoFisher Scientific) and incubation at 70°C for 10 minutes, as recommended by the manufacturer. Electrophoresis was conducted using 1X NuPAGE MOPS SDS running buffer (ThermoFisher Scientific) for 1 hour at 150 V. The samples were stained using Invitrogen Coomassie Simply Blue Safe stain (ThermoFisher Scientific). Equivalent amounts of samples in relation to the number of cells were loaded onto each well. Silver staining was conducted using the Pierce silver stain kit for mass spectrometry (ThermoFisher Scientific) according to the standard manufacturer's protocol. The molecular marker used for Coomassie staining was PageRuler Plus Prestained protein ladder (ThermoFisher Scientific) diluted to 1:10 in 1X NuPAGE LDS buffer (ThermoFisher Scientific). For Silver staining the PageRuler Plus Prestained protein ladder (ThermoFisher Scientific) was diluted to 1:100 in 1X NuPAGE LDS buffer (ThermoFisher Scientific).

### Enzyme-linked immunosorbent assay

Calf skin collagen I (Sigma) was dissolved in 0.1M acetic acid (Fisher Scientific) to a stock concentration of 1 mg/ml. Transparent Corning Costar 96 well flat bottom non-treated polystyrene plates were coated with 0.36 µg/well calf skin collagen I (Sigma) or BSA (Sigma) in ELISA coating buffer (0.19 g Na_2_CO_3_, 0.37 g NaHCO_3_ in 125 ml dH_2_O; pH 9.6). The plates were incubated at 4°C overnight. For washing steps, 200 µl/well PBS with 0.1%Tween 20 (PBST) was used. After coating, the plates were washed twice with PBST and blocked for 1 hour at room temperature with 2% BSA (Sigma) in PBS (200 µl/well). Wells were washed 3 times with PBST and 100 µl/well of samples were added to each well. The plates were incubated at 37°C for 1 hour. If bacteria were used, the samples were incubated under anaerobic conditions. Wells were washed 4 times with PBST. One hundred microliters of the primary antibody (1:1000 dilution in PBS containing 0.05% Tween20 (Sigma) and 1 mg/ml BSA (Sigma)) were added to each well and the plates were incubated at 37°C for 1 hour. Wells were washed 4 times with PBST and 100 µl of an appropriate secondary antibody (1:1000 dilution in PBS containing 0.05% tween 20 (Sigma) and 3% BSA (Sigma)) was added, followed by incubation for 1 hour at 37°C. Wells were washed 4 times with PBST and incubated with a 100 µl of 3,3′,5,5′-Tetramethylbenzidine substrate (Sigma) for 15 minutes. Fifty µl/well of 1M H_2_SO_4_ were added to stop the reaction. Absorbance was measured at 450 nm using a Tecan Infinite M200 Pro microplate reader.

For *C. jejuni* binding and CBP/*C.jejuni* competition assays, *Campylobacter jejuni* monoclonal primary antibody (Bio-Rad) and goat anti-mouse polyclonal secondary antibody (Bio-Rad) were used. For CBP binding assay, Pierce 6x-His Epitope Tag monoclonal primary antibody (ThermoFisher Scientific), and anti-mouse IgG, HRP-linked Polyclonal secondary antibody (Cell Signaling Technology) were used.For whole cell competition assay anti-*Campylobacter jejuni* (PEB1), polyclonal primary antibody (Antibodies-Online) and Goat-anti-rabbit IgG polyclonal secondary antibody HRP conjugate (SAB) were used.

### Binding and competition assay

For binding assay, CBP stock was diluted in PBS. The samples were added to collagen I coated wells. BSA-coated wells were used as negative controls. For *C. jejuni* attachment studies the wells coated with collagen I were incubated with a 100 µl of bacterial suspensions made to an OD_600_ of 1, 0.1 and 0.01 in PBS. The final amounts of *C. jejuni* cells added to each well were 2 × 10^8^ cfu/well, 2 × 10^7^ cfu/well and 2 × 10^6^ cfu/well respectively. BSA-coated wells were used as negative controls. For whole cell competition assay, *L. fermentum* 3872 bacterial suspension was made to an OD_600_ of 0.5 (1 × 10^8^ cfu/ml), 1 (2 × 10^8^ cfu/ml), 5 (1 × 10^9^ cfu/ml), and 9 (2 × 10^9^ cfu/ml) by mixing with *C. jejuni* to have a final bacterial suspension of OD_600_ 0.1 (2 × 10^8^ cfu/ml) in PBS. A hundred microliters of the mixture was added to each well. This resulted in a final ratio of *L. fermentum* 3872 to *C. jejuni* of 1:2, 1:1, 5:1 and 10:1 respectively. For competition assays involving CBP and *C. jejuni*, collagen I or BSA coated wells were incubated with a mixture of 2 µg/well CBP and 2 × 10^7^ cfu/well *C. jejuni* in PBS.

### Agar well diffusion assay

Agar well diffusion assay was used to determine anti*-C. jejuni* activity.^18^ *C. jejuni* suspensions were adjusted to an OD_600_ of 1 in PBS, of which 300 µl were added to 15 ml of soft (0.75%) Mueller-Hinton (MH) agar at 41°C. Soft agar was prepared by mixing MH broth (Fluka) to 0.75% agar (Fluka). The inoculated molten agar was overlaid over 20 ml MH agar. *L. fermentum* 3872 was cultured overnight in M.R.S broth at 37°C under anaerobic condition and filter sterilised using a 0.22 µm filter (Fisher Scientific). The cell-free culture supernatant was either boiled at 100°C for 5 minutes, or the pH was adjusted to that of the M.R.S broth (6.3) using NaOH (Sigma). Four 10 mm wells were cut in the MH agar after inoculating with *C. jejuni*. The wells were filled with 300 µl of one of the following 1) MH broth, 2) cell-free 3872 culture supernatant 3) boiled cell-free 3872 culture supernatant, or 4) cell-free 3872 culture supernatant with adjusted pH.

### Co-Immunoprecipitation and mass spectrometry

Co-Immunoprecipitation (Co-IP) was conducted using Dynabeads Co-Immunoprecipitation kit (ThermoFisher Scientific) to determine collagen I binding proteins expressed by *C. jejuni* 11168H and 81–176. Dynabeads were coated with 15 µg of collagen I (15 µg of collagen per 1 mg of Dynabeads). Standard manufacturer's protocol was followed. *C. jejuni* suspension was made to an OD_600_ of 1 in 20 ml of PBS. The cell suspension was spun down at 3200 g for 10 minutes at 4°C, the supernatant was removed and the bacteria pellet was weighed. Lysis buffer was prepared using 1X IP buffer provided by the Dynabeads Co-Immunoprecipitation kit (ThermoFisher Scientific), 100mM NaCl (Sigma) and 5 µl DNase I (Promega) in dH_2_O. The bacterial pellet was lysed in a 1:9 ratio of cell weight to volume and incubated on ice for 10 minutes. To ensure complete lysis, bacterial lysates were sonicated (Soniprep 159) for 10 cycles with 10 seconds sonication and 30 seconds rest, to complete lysis. After sonication, the samples were spun at 3200 g for 5 minutes. The clarified lysates were incubated at room temperature with 2 mg of collagen I coupled Dynabeads on a rotator (30 rpm) for 1 and 3 hours. Standard manufacturer's protocol was followed for protein elution. Prior to mass spectrometry, Pierce silver stain kit (ThermoFisher Scientific) was used to detect protein bands (see above). Bands were cut out using a scalpel and placed into an Eppendorf tube with 50 µl of dH_2_O. Mass spectrometry was conducted by the Cambridge Center for Proteomics (United Kingdom). Mascot server was used for the identification of proteins using Genbank data of the respective *C. jejuni* strains.

### Statistical analysis

ELISA readings were adjusted by subtracting values of relevant controls. Statistical analysis was conducted using one-way analysis of variance (ANOVA). A P value of < 0.05 was considered as statistically significant. On ELISA graphs the P variances are labeled by stars according to the following scheme: * for 0.005<p≤ 0.05, ** for 0.001<p≤ 0.005 and *** for p≤ 0.001. The vertical bars on the diagrams represent SEMs (standard errors of the mean).

## Supplementary Material

KVIR_S_1362533.zip
