# Supplementary material for: Lactobacillus fermentum 3872 as a potential tool for combatting Campylobacter jejuni infections
Source: Virulence. 2017 Aug 25;8(8):1753–60. doi: 10.1080/21505594.2017.1362533 (PMC5810503; doi:10.1080/21505594.2017.1362533)
Supplement: KVIR_S_1362533.zip [file kvir-08-08-1362533-s001.zip › KVIR_S_1362533_Fig2.docx]

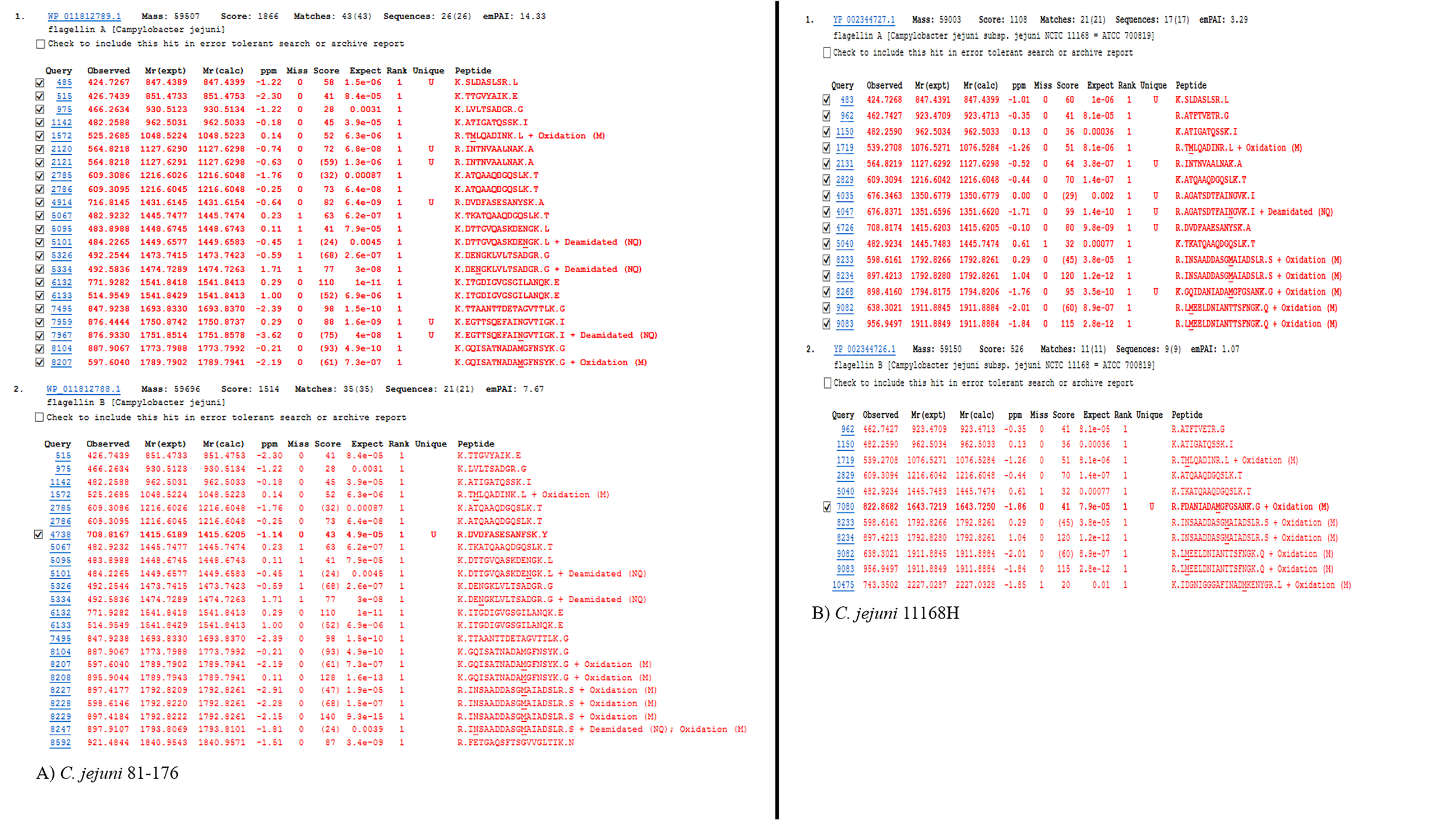


**Figure S2**

Screenshots of Mascot analysis of LC MS/MS output data; the diagram shows predominant hits for a 65 kDa collagen binding protein detected in *C. jejuni* strains 81-176 (A), 11168H (B).
