# Supplementary material for: Lactobacillus fermentum 3872 as a potential tool for combatting Campylobacter jejuni infections
Source: Virulence. 2017 Aug 25;8(8):1753–60. doi: 10.1080/21505594.2017.1362533 (PMC5810503; doi:10.1080/21505594.2017.1362533)
Supplement: KVIR_S_1362533.zip [file kvir-08-08-1362533-s001.zip › KVIR_S_1362533_Fig1.docx]

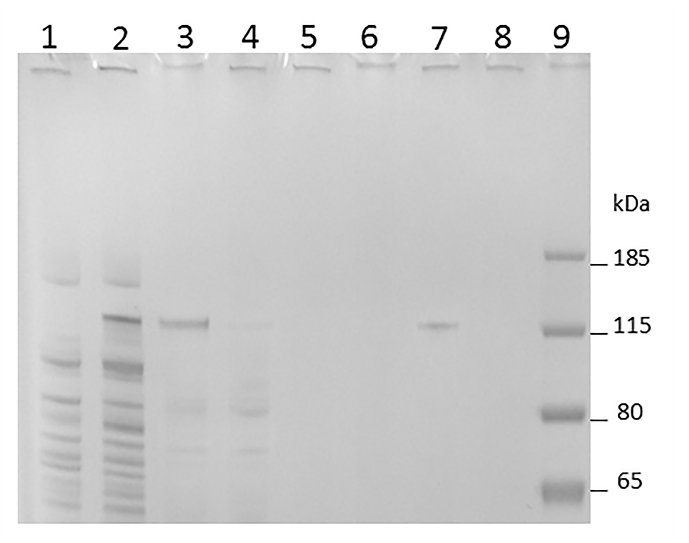


**Figure S1**

Coomassie staining of the recombinant CBP: 1, lysate before induction; 2, lysate after 3 hours induction; 3, clarified lysate; 4, flowthrough; 5, wash; 6, eluate 1; 7, eluate 2; 8, eluate 3; 9, pre-stained ladder (Page ruler plus).
